# The quality of sleep and digestion in cerebral palsy depends not only on the level of functional independence

**DOI:** 10.1590/1806-9282.20231637

**Published:** 2024-05-20

**Authors:** Walter Michael Strobl, Carla Alexandra Scorza, Fulvio Alexandre Scorza, Josef Finsterer

**Affiliations:** 1 Danube University for Continuing Education Krems and MOTIO, Department of Health Sciences, Medicine and Research – Vienna, Austria.; 2 Universidade Federal de São Paulo, Paulista School of Medicine, Discipline of Neuroscience – São Paulo (SP), Brazil.; 3 Neurology and Neurophysiology Center – Vienna, Austria.

Dear Editor,

We read with interest Gunaydin and Tuncer's article, which is a cross-sectional, single-center, observational study on the association of the levels of functional independence with sleep and constipation in 60 children aged 4–18 years with cerebral palsy (CP) carried out between September 2021 and April 2022^1^. It was found that 46.7% of the cases corresponded to level III on the Gross Motor Function Classification System (GMFCS), 35% to level IV, and 18.3% to level V^1^. There was a negative correlation between the functional independence measure (FIM) for children and pediatric sleep questionnaire (PSQ) and between FIM for children and the constipation severity scale (CSS)^1^. It was concluded that low levels of functional independence correlate with poorer sleep and constipation^1^. The study is impressive, but some points require discussion.

The major limitation of the study is that alternative factors that influence sleep quality and digestion were not adequately included in the analysis. Digestion depends not only on the level of mobility, but also on the type of diet, level of hydration, sympathetic parasympathetic balance, psychiatric status, stress level, current medications, body mass index, and sleep quality. Therefore, we should know the diet of the 60 patients included during the study period, as well as the type and amount of fluids in relation to their body weight. The composition, quality, and quantity of food largely determine the function of the intestines. It is also important to know the level of exogenous and endogenous stress to which the included patients were exposed. Digestion also depends on a person's temperament. Other factors that affect digestion include mood, drive, and threat level. Therefore, it would have been advisable to administer depression and anxiety scales to all enrolled patients.

In addition, there are a number of comorbidities that can affect digestion. Although patients with certain comorbidities or medical conditions (surgery to improve intestinal health, chronic infectious bowel disease, congenital intestinal anomalies, treatment with botulinum toxin in the last 6 months, uncontrolled seizures, constipation in the last 6 months, cardiopulmonary disease) were excluded, multiple comorbidities were not included in the list of exclusion criteria. These include gastritis, diabetes, hypo-/hyperthyroidism, hypo-/hypercorticism, and hypo-/hyperaldosteronism^2^.

Sleep quality may depend not only on the level of functional mobility but also on several other factors. These include the level of noise in the bedroom at the beginning and during sleep, the time of last food/liquids intake, the presence/absence of pain during the night, the presence/absence of restless legs, concomitant medications taken at night, degree of sympathetic/parasympathetic activation, presence/absence of nocturia, presence/absence of nocturnal seizures, temperature in the bedroom, lighting conditions in the bedroom, type of daytime experiences and their processing during sleep, air quality in the bedroom (level of pollution, urban or rural area), time of going to bed, and the underlying etiology of CP, i.e., the location of the cerebral lesion^3^. Of particular interest is whether the sleep/wake rhythm center was affected. Therefore, sleep quality should also be correlated with cerebral imaging findings and the EEG.

Additional limitations include that the group size was small, the design was single center, and test-retest reliability was not assessed.

In summary, the interesting study has limitations that put the results and their interpretation into perspective. Clarifying these weaknesses would strengthen the conclusions and could improve the study. Despite all possible objections, we agree that all necessary measures must be taken to achieve the highest possible level of functional mobility and to enable the affected CP individuals to have the highest possible level of independence.

## Data Availability

Data that support the findings of the study are available from the corresponding author.
